# Functions of heteranthery and enantiostyly for wing pollination by pollen-collecting bees in *Dilatris ixioides* (Haemodoraceae)

**DOI:** 10.1093/aob/mcaf189

**Published:** 2025-08-23

**Authors:** Steven D Johnson, Jeremy J Midgley, Nicola Illing

**Affiliations:** Centre for Functional Biodiversity, University of KwaZulu-Natal Pietermaritzburg 3209, South Africa; Department of Biological Sciences, University of Cape Town Cape Town 7701, South Africa; Department of Molecular and Cell Biology, University of Cape Town Cape Town 7701, South Africa

**Keywords:** Carpenter bees, floral evolution, mirror-image flowers, monomorphic enantiostyly, nectar, pollen, pollination syndrome, *Xylocopa*

## Abstract

**Background and Aims:**

Evolutionary floral modifications that enable plants to exploit particular body parts of animals for pollen transfer are considered a key contributor to the angiosperm radiation. Species of *Dilatris* (Haemodoraceae) have an unusual combination of floral traits including a centrally positioned stamen with a large anther flanked by two stamens with smaller anthers (heteranthery) and styles that are deflected either to the right or left on flowers of the same plant (monomorphic enantiostyly). We investigated the pollination functions of these traits in *D. ixioides* to better understand their evolution.

**Methods:**

We quantified floral traits including morphology, pollen production by different anthers and spectral reflectance of various organs. We also assessed the identity and pollen loads of flower visitors and their behavioural responses to anther removal, as well as the levels of self-compatibility and pollinator dependence of *D. ixioides*.

**Results:**

Left- and right-styled flowers of *D. ixioides* alternate consistently along inflorescence branches (pendulum symmetry). They do not produce nectar and were found to be pollinated primarily by female carpenter bees that collect pollen from the large central anther. The central anther is yellow, has spectral properties similar to those of pollen and produces more than twice as many pollen grains as do each of the smaller lateral anthers, which are red. Flowers with the central anther experimentally removed were ignored by bees. Pollen is transferred via the bee’s wings as they beat against the deflected lateral anthers and stigma while the bees grasp the central anther. Plants are self-compatible but reliant on pollinator visits for seed production.

**Conclusions:**

The absence of nectar production and development of a large central pollen-rewarding anther in combination with laterally deflected anthers and styles in *D. ixioides* are functional components of an effective and novel system of wing pollination by female bees.

## INTRODUCTION

Nectar production has been gained and lost numerous times in the evolution of the flowering plants (Vogel, 1978; Renner, 2006, p. 754; Johnson et al., 2013). One of the most common reasons for loss of nectar production is floral specialization for pollination by pollen-collecting insects, particularly female bees (Buchmann, 1983). It has been estimated that approximately 6–10 % of plant species, representing at least 52 families, offer only pollen as a reward (Vogel, 1978; Buchmann, 1983). The provision of pollen as the sole reward is often accompanied by modifications of stamen functions. These modifications can include the development of poricidal anthers that require specialized vibration (‘buzz-pollination’) from flower visitors to release concealed pollen (Vallejo-Marin and Russell, 2024), as well as heteranthery, the subdivision of stamens between those with ‘feeding anthers’ that provide the pollen that serves as a reward and those with ‘pollinating anthers’ that provide the pollen that is transferred to stigmas, leading to fertilization of ovules (Luo et al., 2008; Vallejo-Marin et al., 2009; Barrett, 2021). Feeding anthers are often located in the centre of the flower and are more conspicuous than pollinating anthers, and this is believed to focus the attention of pollen-collecting insects on feeding anthers (Vallejo-Marin et al., 2009), thereby minimizing deleterious collection of pollen from the pollinating anthers and resulting gametic wastage.

Floral organs with a female function are also often modified in pollen-rewarding flowers (Buchmann, 1983). For example, the style is often deflected away from the centrally positioned feeding anthers, and in some cases, referred to as enantiostyly, the style is deflected either to the left or right in mirror-image fashion (Barrett, 2002; Jesson and Barrett, 2003; Barrett and Fairnie, 2024; Robertson et al., 2025). The advantages of enantiostyly can include reduction of geitonogamy (Jesson and Barrett, 2002b), avoidance of physical damage (Dulberger, 1981) and utilization of lateral insect body parts, such as their wings, for pollen transfer (Johnson et al., 2023).

Although heteranthery and enantiostyly are often part of the floral syndrome of pollen-rewarding plants (Buchmann, 1983; Barrett, 2002, 2021; Barrett and Fairnie, 2024), they can also be found in some nectar-rewarding species (Dellinger et al., 2021; Minnaar and Anderson, 2021; Johnson et al., 2023). To understand the function of these traits it is therefore necessary to conduct field studies of pollination mechanisms, including responses of animals to floral rewards and the transfer of pollen on their body parts. High-speed videography, for example, has provided important new data about the function of stamen modifications in pollen-rewarding flowers (Woodrow et al., 2024). This approach is a valuable method for understanding the function of enantiostyly in plants that place pollen on the wings of flower visitors (Minnaar and Anderson, 2021; Johnson et al., 2023), as the short duration of visits and the rapid wingbeats of insects make it difficult when using direct human observations to ascertain whether there is contact between rapidly beating wings and stigmas.

Floral evolution in the Haemodoraceae subfamily Haemodoroideae is of particular interest given the global distribution of the lineage (Simpson, 1990; Hopper et al., 2009; Pellegrini et al., 2020) and the occurrence of both monomorphic enantiostyly (with left versus right style deflection varying within an inflorescence) and dimorphic enantiostyly (left versus right style deflection on different plants in a population) (Jesson and Barrett, 2003). Dimorphic enantiostyly has been confirmed in studies of the South African genera *Wachendorfia* and *Barberetta* (Ornduff and Dulberger, 1978; Johnson et al., 2023), while Jesson and Barrett (2003) inferred from taxonomic descriptions and their own observations that monomorphic enantiostyly occurs in *Dilatris* in South Africa, *Schiekia* and *Xiphidium* in central and southern America, *Lachnanthes* in North America and *Haemodorum* in Australia. Although relationships among the genera in subfamily Haemodoroideae are not yet fully resolved (Hopper et al., 2009), the available evidence indicates a transition from monomorphic to dimorphic enantiostyly in this lineage.

There is notable variation in the arrangement of stamens in the Haemodoroideae. *Wachendorfia* and *Barberetta*, for example, have two stamens deflected away from the style and one deflected in the same direction as the style and no anther dimorphism (Ornduff and Dulberger, 1978; Johnson et al., 2023). In *Schiekia* the median anterior stamen is deflected opposite to the style and the other two stamens are reduced (Pellegrini et al., 2020). In *Xiphidium* the stamens are all of a similar size and form a whorl in the centre of the flower (Buchmann, 1980; Pellegrini et al., 2020). In *Cubanicula* there are two short stamens in the centre of the flower and a single elongated stamen, which is deflexed opposite to the style (Pellegrini et al., 2020). In *Lachnanthes* there are three stamens radially organized around the style (Simpson, 1990). In *Dilatris* there is one centrally positioned stamen with a large anther flanked by two deflected stamens with smaller anthers (Jesson et al., 2003; Manning and Goldblatt, 2017). An intriguing and novel feature of heteranthery in *Dilatris* is the difference in colour between the yellow central anthers and the red lateral anthers. The deflected lateral anthers are expected to have a greater role than the central anther in pollen transfer to the deflected stigma and thus to exhibit greater spatial reciprocity with the style. The significance of these various stamen arrangements in the Haemodoroideae is not yet fully understood because of incomplete information about the pollination and sexual systems in the lineage. Available evidence suggests that deflected styles and stamens in the subfamily are associated with various mechanisms of pollen placement on lateral body parts of insects, including their wings in some cases (Minnaar and Anderson, 2021; Johnson et al., 2023).

The reproductive biology of *Dilatris* has not previously been studied, aside from one attempt to measure geitonogamy using stained pollen grains (Barrett et al., 2000). Simpson (1993) noted that *Dilatris* has vestigial supralocular septal nectaries that appeared to be non-functional and suggested that observations of pollination in the genus ‘might help elucidate the effective loss of nectaries in that genus’. Related genera such as *Haemodorum*, *Schiekia*, *Lachnanthes*, *Wachendorfia* and *Barberetta* are considered to be nectar-producing and have well-developed nectaries (Simpson, 1993; Pellegrini et al., 2020). However, *Xiphidium* lacks nectaries and has a pollen-rewarding pollination system involving vibratile (buzz pollination) collection of pollen by bees (Buchmann, 1980). Available evidence suggests that *Cubanicula* and *Pyrrorhiza*, which are closely related to *Xiphidium*, are also nectarless and pollen-rewarding (Simpson, 1993; Pellegrini et al., 2020). The unique combination of stamen arrangement, dimorphism of anthers, and vestigial nectaries in *Dilatris*, which is considered closely related to *Lachnanthes* (Hopper et al., 2009), suggested to us that this genus may represent an independent shift from nectar to pollen rewards in the subfamily Haemodoroideae.

The aim of this study was to assess the pollination functions of heteranthery and enantiostyly in *Dilatris* in order to clarify the evolutionary basis of these traits, which are found in several plant lineages (Barrett et al., 2000; Vallejo-Marin et al., 2009; Barrett, 2021; Barrett and Fairnie, 2024), and thus to shed more light on floral evolution in these lineages. We predicted that *D. ixioides* has a system of wing pollination involving insects that collect pollen from the central anther. We specifically investigated the identity and behaviour of flower visitors and their potential for pollen transfer; floral morphology, including the distribution of left- and right-deflected styles within and among plants and the three-dimensional reciprocity of the style and anthers among morphs; pollen production and size in central and lateral anthers; spectral reflectance of flower parts, including pollen; and the sexual system, including self-compatibility and capacity for autogamy.

## METHODS

### Study genus and species

**Fig. 1.**
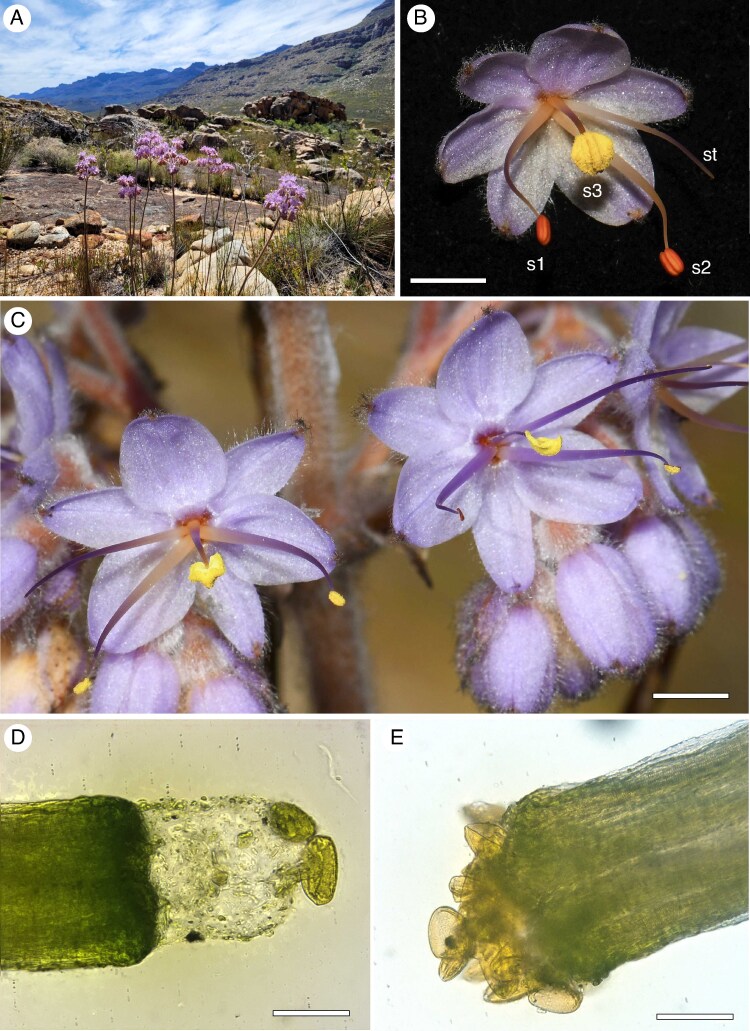
Habitat and morphology of *D. ixioides*. (A) Flowering plants in fynbos vegetation at the study site in the Cederberg mountains. (B) Left-styled flower during anthesis showing the position of the styles (st) and the numbering convention we used for the different stamens (s1–s3). Note the red colour of the unopened lateral anthers. (C). Left- and right-styled flowers on the same inflorescence. (D) stigma showing the exudate at the tip. (E) Stigma with close to the maximum number pollen grains that can be accommodated. Scale bars: B–C = 5 mm, D–E = 50 µm.

*Dilatris* is a small genus of four species confined to the Cape Floristic Region of South Africa (Manning and Goldblatt, 2017) where they occur in fynbos shrubland vegetation (Fig. 1A). The flowers (arranged in a complex bifurcating or trifurcating helicoid cyme) are characterized by a short central stamen with a large anther and two longer laterally deflected stamens with smaller anthers, as exemplified by *D. ixioides* (Fig. 1B). In three of the species the lateral anthers are red while the central anther is yellow (Fig. 1B). The genus is characterized by monomorphic enantiostyly with both left- and right-styled flowers on the same plant. Anther dimorphism and enantiostyly are strongly developed in *D. ixioides* (Fig. 1C), *D. corymbosa* and *D. viscosa*, but weakly developed and barely discernible in *D. pillansii* (Manning and Goldblatt, 2017). The ovary has three locules, each with a single ovule.

*Dilatris* has close phylogenetic affinities with *Haemodorum* in Australia and *Lachnanthes* in North America (Hopper et al., 2009; Pellegrini et al., 2020). Simpson (1993) noted that these three genera share the synapomorphies of an inferior ovary, discoid seeds and pollen with one-layered exine.

*Dilatris ixioides* is the most widely distributed and common species in the genus and is found from the Cederberg mountains in the north through to the Outeniqua mountains the east of the L-shaped Cape Floristic Region. As a result of its relative abundance we focused our studies on this species.

### Study site

Our experiments and observations were conducted in several large populations of *D. ixioides* consisting of thousands of individual plants at ‘die Drift’, a private farm (32°41.491ʹS, 19°16.787ʹE, elevation: 662 m) in the Cederberg Mountains (Fig. 1A). Fieldwork was conducted in October–November in 2023 and 2024. Voucher specimens from these populations were deposited in the Bews Herbarium (NU) at the University of KwaZulu-Natal (Johnson s.n.).

### Pollinators and their behaviour

We conducted pollinator observations from 0700 h until 1500 h for a total of 7 days. Three observers patrolled the population and noted flower visitors and collected representative samples and measured their body and wing lengths to the nearest 0.1 mm using digital callipers. Subsamples of pollen were removed from the insects and compared to reference slides of pollen of *D. ixioides*. The proportion of *D. ixoides* pollen grains in samples taken from the wings and scopae of bees were compared using a binomial generalized linear mixed effects model (GLMM) with an events/trials structure and bee identity treated as a random effect. We also added an observation-level random effect to control for overdispersion (Bolker, 2015). Unless stated otherwise, all statistical analyses were conducted using SPSS 28 (IBM Corp.).

In addition to direct observations, we also used four motion-activated cameras (Bushnell Nature View 119740) to make additional records of flower visitors. To understand the sequence of pollinator behaviour on flowers, we filmed insect flower visitors using high-speed videography at 120 frames per second in HD format (1020 × 1080 pixels) using a Sony AX53 camcorder. The footage was slowed down 50 times (2 % of the original speed) to enable detailed elucidation of the behaviour of flower visitors, including active pollen collection from the anthers, contact of their body parts with the anthers and the stigma, and the duration of these events. Slowing down the videos also allowed for additional qualitative assessment of pollen loads (as small or large) on the wings of flower visitors. To determine whether flowers produce nectar as a reward, we observed whether insect visitors probed the base of the flowers with their tongues. We also excluded pollinators from flowers for 24 h using fine mesh bags and then ascertained if any nectar from the base of these flowers could be drawn into fine 5 µL pipettes, which were subsequently examined under a dissecting microscope for evidence of liquid uptake.

### Emasculation experiment

We experimentally tested whether the presence of the large yellow central anther is an essential cue for pollinator visits. To do this we removed the central anther from half of the open flowers on two plants of *D. ixioides*. Each emasculated flower was paired with an intact flower (*n* = 19 flower pairs on plant 1 and 15 flower pairs on plant 2). We monitored the two inflorescences using continuous video recorded with two Mobius maxis cameras (Huizhou tuopu xunshi Technology Co., Ltd, Huizhou City, PRC) from 0830 h to 1800 h for 2 days. For each flower visitor, we recorded the number of intact and emasculated flowers that were visited. Data were analysed using a binomial GLMM with an events/trials structure for the flower choices made by each individual and included insect species as a fixed factor and Sidak tests for post-hoc comparison of means. The model was adjusted for overdispersion and we used marginal means and their 95 % confidence intervals to assess whether insect species showed a preference for intact flowers (means with confidence intervals that are fully above or below 50 % represent significant preferences). Because binomial GLMMs require some variance, we had to substitute a single data value for each bee group to allow the statistical model to converge and this renders the test more conservative.

### Floral traits

#### Floral development, dimensions and enantiostyly

We measured the total number of flowers produced per plant by counting flower scars (base of each pedicel) on dried inflorescences of 10 plants. We also measured the number of flowers that were simultaneously open (the display size) for 113 inflorescences (one per plant) of arbitrarily selected plants and counted the number of inflorescences produced per plant from a sample of 63 arbitrarily selected plants that were photographed. We included only flowers with intact anthers as the anthers abscise in older flowers while the perianth remains intact without closing and dries out on the plant. To establish the rate of flowering opening, we removed open flowers from 17 inflorescences and counted the number of flowers that reached anthesis on the following day.

To ascertain the developmental sequence of stylar deflection in *D. ixioides*, we photographed buds at different stages of development, and recorded style orientation in the sequence of flowers on inflorescence branches on five plants. Morphological traits that were measured included flower width, the lateral stamen length, the distance between the lateral anthers, the distance from the lateral anther to the central axis of the flower, the length and width of the lateral anthers, the central stamen length, the central anther length and width. We also measured the horizontal orientation of flowers (angle relative to the horizontal plane) because we surmised that the helicoidal pattern of flower opening would require that the flowers maintain a horizontal orientation across the lateral anthers for a wing pollination mechanism to function. To measure flower orientation we positioned the edge of a Samsung S23 FE phone across the lateral stamens and used the android ‘clinometer’ app developed by Plaincode (https://www.plaincode.com/; last accessed on 4 January, 2024). To compare morphological traits among left- and right-styled flowers and among central and lateral stamens we used GLMMs with a Gaussian error structure and identity link function. Plant and flower identity were treated as random effects.

To determine the degree of three-dimensional reciprocity in positioning of styles and anthers, we photographed flowers from the front and from the side and with a steel ruler for scale. We used ImageJ to calibrate a measurement scale for each photograph and measured the *x*, *y* and *z* coordinates of each floral organ relative to the base of the flower. We plotted the multidimensional spatial coordinates of the organs and calculated the standardized total inaccuracy metric (Armbruster et al., 2017) that was adapted for multidimensional data in the R package FlowerMate (Simón-Porcar et al., 2024). Standardized total inaccuracy values (3DM^2^STI) are based on equally weighted overall departure from optimal positioning and imprecision (within population variance) for both morphs in relation to the average Euclidian distance of each floral organ from the central base of the flower. A value of 0 (i.e. zero inaccuracy) would indicate perfect matching of reciprocal organs in multidimensional space and lack of variance among individuals.

#### Pollen production and pollen size

Inflorescences with mature flower buds were placed indoors with the cut stalk in water. Once flowers had opened, we removed individual anthers and counted pollen grains in the lateral anthers versus the central anther using two methods. For the first method, individual anthers from 10 flowers (each from a different plant) were dabbed onto blocks of fuschin gel, which was melted under a cover slip, allowing all of the pollen grains to be counted using scans of the slide using a compound microscope at 400× magnification. For the second method, which is less time-consuming, individual anthers from 27 flowers (each from a different plant) were placed into individual 1 mL Eppendorf tubes and 0.5 mL of 70 % ethanol and 10 µL of 1 % methylene blue solution was added. We took a 10 µL subsample from each Eppendorf tube while agitating it using a mechanical shaker and counted all of the pollen grains using a compound microscope at 40× magnification and then multiplied the value by the ratio of the original volume to the subsample. To assess whether a single subsample was sufficient to estimate pollen production, we took a second subsample from 19 of the 71 Eppendorf tubes. There was a very strong statistical correlation between the first and second subsamples (Pearson correlation = 0.96, *P* < 0.0001) and the use of a single subsample was therefore deemed sufficient.

To compare pollen production between the central anther and lateral anthers we used GLMMs with a negative binomial error structure and log link function. Flower identity was treated as a random effect. Initial analyses showed that the pollen production of lateral anthers did not differ overall according to left and right positions (*F*_1,21_ = 0.18, *P* = 0.67) or left- versus right-styled flower types (*F*_1,15_ = 0.07, *P* = 0.79) and that there was also no significant interaction among these factors (*F*_1,21_ = 0.40, *P* = 0.53). Flower type also had no effects on overall pollen production by anthers (*F*_1,20_ = 0.15, *P* = 0.69) or on the difference in pollen production between central and lateral anthers (*F*_1,40_ = 0.005, *P* = 0.94). We therefore dropped flower type and its interactions from subsequent models of pollen production and focused only on the overall differences in pollen production between central and lateral anthers.

We measured the length and width of 46 pollen grains from central anthers and 51 pollen grains from lateral anthers taken from 10 plants. To compare pollen size between the central anther and each lateral anther we used GLMMS with a Gaussian error structure and identity link function and with plant identity treated as a random effect.

#### Spectral reflectance

Spectral reflectance of upper, lateral and lower tepals, lateral and central anthers, as well as pollen grains from the lateral and central anthers was measured in the 300–700 nm range using an Ocean Optics S2000 spectrometer, as described by Johnson and Andersson (2002). To measure the spectral reflectance of pollen, we dabbed freshly dehisced anthers onto clear Sellotape until it was uniformly covered with pollen. The use of a small-diameter (200 µm bundle) fibre-optic reflectance probe made it possible to obtain reflectance measurement from these small objects.

#### Stigma pollen loads

To assess the dimensions of stigmas and their pollen loads, we removed styles from single flowers taken from each of 57 plants in the field and mounted them on slides in fuchsin gel. They were then examined with a compound microscope at 100× magnification and the stigma dimensions for 13 of these flowers were measured using the measuring tool in Zeiss Zen software (Zeiss, Oberkochen, Baden-Württemberg, Germany) and the number of pollen grains per stigma was counted for the 57 flowers. We established the identity of the pollen grains by comparison to reference slides of pollen from anthers of *D. ixioides*.

#### Controlled pollination experiments

We conducted controlled hand-pollination experiments to determine the compatibility system and pollinator dependence of *D. ixioides.* Mature flowers buds on 28 plants in a single population were enclosed with fine insect-proof netting. Once flowers on these plants had opened, they were assigned to one of four treatments: unmanipulated, to test for autonomous self-fertilization, manually self-pollinated to test for self-compatibility, manually cross-pollinated with pollen from the lateral anthers and manually cross-pollinated with pollen from the central anther. Each plant received all treatments in a ‘split plot’ design and a total of 175 flowers were assigned to the treatments, meaning that each plant typically had several flowers per treatment group. We scored these flowers for fruit formation and dissected fruits to establish their number of ovules and seeds 6 weeks after hand-pollinations when the fruits were swollen. We also scored natural fruit and seed set for 137 flowers on a further 10 plants that were at the same stage of development as the plants used in the controlled hand-pollinated experiments.

To compare the proportion of flowers that set fruit and the proportion of ovules that set seed among the treatment groups, we used binomial GLMMs with plant identity treated as a random effect and treatment as a fixed factor. We analysed the proportion of flowers that set fruit containing seeds and the proportion of ovules that developed into seeds. Post-hoc comparison of means was based on the sequential Sidak method.

## RESULTS

### Pollinators and their behaviour

We recorded 321 visits to flowers of *D. ixioides* involving 180 insect individuals (Table 1). Flowers of *D. ixioides* were visited by several pollen-collecting insects, including carpenter bees (*Xylocopa*: Xylocopinae; Apidae), digger bees (*Amegilla*: Anthophorini; Apidae), monkey beetles (*Petitrichia* and *Burmeistoplia*: Hopliini; Scarabaeidae) and nose flies (*Cosmina*: Rhiniinae; Calliphoridae). These insects mainly collected or fed on pollen from the central anther. They only very rarely collected pollen from the lateral anthers and never extended their tongues and probed the base of the flower in a manner that would suggest nectar feeding. No traces of nectar could be extracted from the flowers using micropipettes.

**Table 1.** Dimensions of insect visitors to flowers of *Dilatris ixioides* and their behaviour based on direct observations and video recordings.

Taxon	*N*_vis_	*N*_ind_	Body length (mm)	Wing length (mm)	Records of anther used for pollen feeding	
					Centre	Lateral
Apidae						
*Xylocopa rufitarsus*	172	82	17.4 ± 0.27	16.2 ± 0.48	112	0
*Xylocopa caffra*	37	24	22.9 ± 0.32	22.3 ± 0.48	17	2
*Apis mellifera*	2	1	c. 12	c. 9	1	1
*Amegilla* sp.	20	4	c. 14	c. 12	20	0
Halictidae						
Species 1	9	6	c. 5	c. 5	8	1
Calliphoridae						
*Cosmina* sp.1	51	37	8.4 ± 0.29	6.1 ± 0.43	27	7
Scarabidae						
*Burmeistoplia scutellaris*	11	11	10.6 ± 0.27	–	3	1
*Peritrichia* sp. 1	14	13	8.1 ± 0.24	7.7 ± 0.62	5	0
*Peritrichia* sp.	4	3	8.3 ± 0.51	–	2	0

*N*_vis_ = flower visits recorded, *N*_ind_ = individuals observed or captured. Values are means ± s.e. Values with a prefix of c. are estimates based on specimens that were not captured on flowers of *D. ixioides*.

**Fig. 2.**
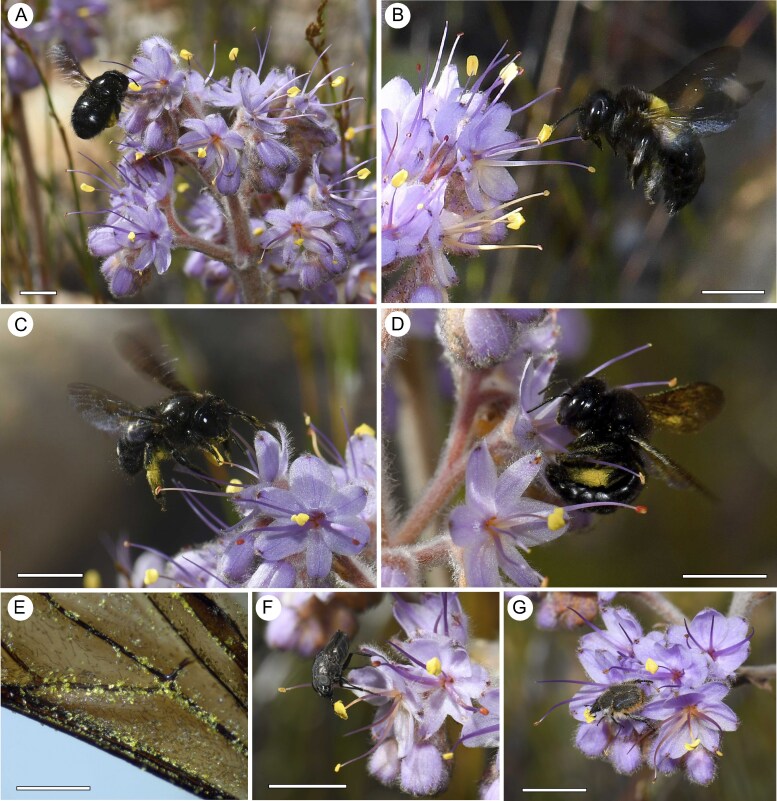
Insect visitors to flowers of *D. ixioides*. (A) Carpenter bee *Xylocopa rufitarsus* approaching an inflorescence. (B) *Xylocopa caffra* touching the central anther with its antennae. (C) *X. rufitarsus* about to grasp the central anther. (D) *X. rufitarsus* grasping the central anther with large deposits of pollen visible on its wings. (E) Close-up of *Dilatris* pollen on the wing of *X. rufitarsus*. (F) Nose fly (*Cosmina* sp.) feeding on pollen from the central anther. (G) Hopliine beetle (*Peritrichia* sp.) feeding on pollen from the central anther. Scale bars: 10 mm, apart from E = 1 mm.

**Fig. 3.**
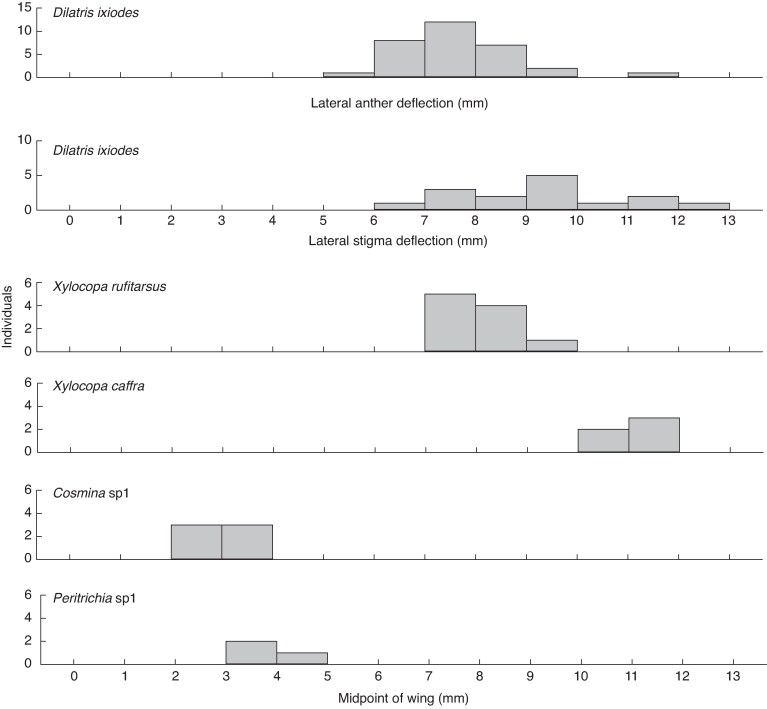
Matching of dimensions of flowers of *D. ixioides* and the wings of insect flower visitors. Wings of the carpenter bee *X. rufitarsus* most closely match the lateral deflection of anthers and the stigma of *D. ixioides*.

Carpenter bees were the most common visitors to flowers of *D. ixioides* (Fig. 2A–D, Table 1). Their wings were densely coated in pollen (Fig. 2D, E) and high-speed videography (Supplementary Data Video S1, Fig. S1) revealed that their wings beat against the lateral anthers and the stigmas while they collect pollen from the central anther. The most common carpenter bee visitor was *Xylocopa rufitarsus* (Fig. 2A, C, D). The midpoint of the wings of this bee species corresponds very closely to the lateral deflection of outer stamens and the style of *D. ixioides* (Fig. 3). The larger carpenter bee species *X. caffra* (Fig. 2B) also visited the flowers and collected pollen from the central anther (and occasionally from lateral anther), but due to its large size it did not contact the lateral anthers and style while feeding from the central anther as often as did *X. rufitarsus*. All the captured carpenter bees were females, which is consistent with their pollen-collecting behaviour, and we confirmed the presence of *D. ixioides* pollen on the wings of all specimens of *X. rufitarsus*. Fuchsin gel swabs taken from the wings of nine individuals of *X. rufitarsus* contained a median of 880 *D. ixioides* pollen grains per sample, but this was only a small fraction of the total pollen loads on their wings. Pollen of *D. ixioides* made up 89.7 ± 1.4 % of all pollen grains in swabs taken from the wings versus 60.2 ± 3.7 % in swabs taken from the scopae (*F*_1,16_ = 4.92, *P* = 0.04).

Carpenter bees visited a mean (± s.e.) of 2.3 ± 0.29 flowers per inflorescence (out of the ∼13 flowers open simultaneously, see below). The mean duration of visits to individual flowers was 1083 ± 105 milliseconds (ms) and these visits involved a highly stereotyped behavioural routine. They approached the central stamen and stroked it with both of their antennae for ∼113 ± 8.9 ms. The antennae of carpenter bees were usually coated in pollen from this anther stroking/sniffing behaviour. They then either moved to another flower or proceeded to grasp the central anther between their legs while hovering and actively collect pollen for ∼1079 ± 132 ms. They usually hovered throughout the pollen collection phases, but sometimes momentarily stopped beating their wings while grasping the filament of the central stamen in their jaws (Fig. 2), allowing them to use all their legs for grooming. The wings of carpenter bees beat against the anthers and style for ∼472 ± 56 ms during each visit. The wing contact during flower approach was ∼232 ± 20 ms and during flower departure was ∼272 ± 71 ms.

Carpenter bees appeared to be the most important pollinators of *D. ixioides*, based on their relative abundances (Table 1), morphological fit to the flowers (Fig. 3), large pollen loads (Fig. 2D, E) and behaviour, which included regular contact with the anthers and styles (Fig. 2A–D). *Amegilla* bees were the only other insects that made occasional wing contact with the lateral anthers and style and may play a small role as secondary pollinators. Smaller bees, flies and beetles (Fig. 2F, G) very rarely made contact with the reproductive parts of the flowers and their wingspans are much too small to contact the lateral anthers when flying into the flower (Table 1, Fig. 3). Furthermore, hopliine beetles fold their wings under the elytra before landing, making them particularly unsuitable agents of wing pollination.

### Emasculation experiment

**Fig. 4.**
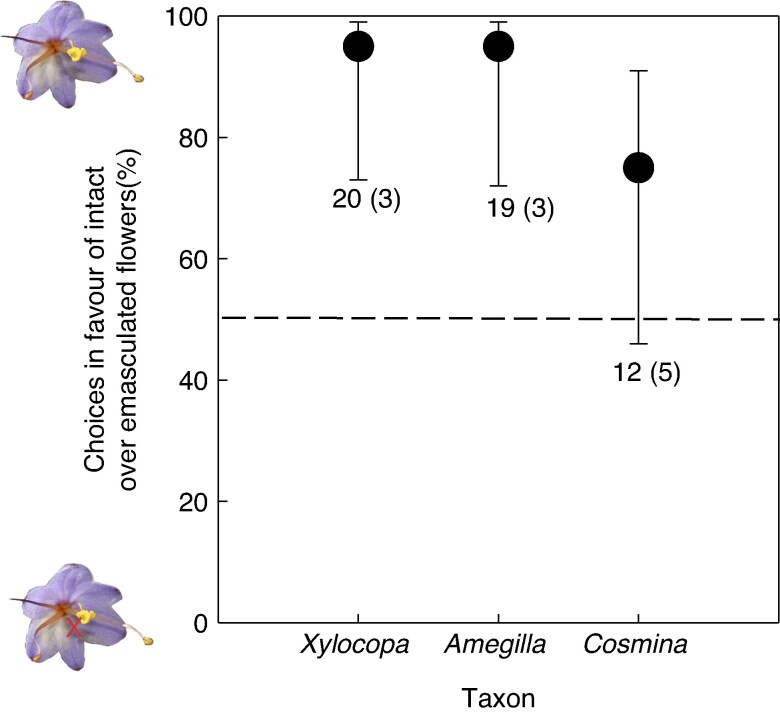
Mean (±95 % confidence intervals) preferences of insects for intact flowers when offered pairwise choices between intact flowers and flowers from which the central anther was removed. Sample sizes are the number of choices and the number of insect individuals in parentheses. Means with confidence intervals that do not overlap 50 % represent significant preference for intact flowers.

In choice experiments, *Xylocopa* and *Amegilla* bees exclusively visited intact flowers with a central anther and ignored neighbouring emasculated flowers without a central anther. The 95 % confidence intervals for the mean choice for the bees exceeded 50 %, indicating a significant preference of these insects for intact flowers (Fig. 4). *Cosmina* flies also mainly visited intact flowers, but this was not statistically significant (Fig. 4).

### Floral development, dimensions and enantiostyly

The mean (± s.e.) number of flowers produced per inflorescence of *D. ixioides* was 85.9 ± 28.7. The number of flowers with intact anthers that were open simultaneously on each inflorescence was 12.1 ± 0.65 and plants produced a mean of 3.6 ± 0.32 inflorescences in each season. The mean number of flowers that reached anthesis per day per inflorescence was 5.2 ± 0.49. Anthers abscised from flowers approximately 48 h after anthesis.

Styles were found to be deflected to the left or to the right in a consistent alternating developmental sequence among flowers on inflorescences of each plant (Supplementary Data Fig. S2). This is an example of the developmental pattern known as pendulum symmetry (Goebel, 1928; Tucker, 1998), also referred to as pendulum asymmetry by Barrett (2002). The lateral stamens with red anthers and the style are folded over the central stamen with a yellow anther in immature *D. ixioides* flower buds (Supplementary Data Fig. S2). The lateral anthers and style emerge first as the flower opens. The central anther initially faced downwards and then reorientates 180° to an upward orientation as the flower bud opens and then dehisces. The central yellow anther was the first to dehisce, followed by the lateral red anthers (Supplementary Data Fig. S2). It therefore seems likely that flowers that attract bees to their dehisced central anther can receive pollen (i.e. have a female function) prior to exporting pollen from their later-maturing lateral anthers.

The style length of sampled flowers was 16.1 ± 0.26 mm and did not differ according to style orientation (χ^2^_1_ = 0.09, *P* = 0.75). The mean stigma diameter was 150.6 ± 3.4 µm. Overall flower width was 17.3 ± 0.59 mm and did not differ according to style orientation (χ^2^_1_ = 1.67, *P* = 0.19). Measured in a clockwise direction, the mean (± s.e.) angle across the lateral anthers of left-styled flowers showed a slight tilt to the left (−4.7 ± 3.4°), whereas right-styled flowers were tilted slightly to the right (12.8 ± 3.9°). This difference was significant (*F*_1,42_ = 11.43, *P* = 0.002). The overall mean angle for all flowers was 4.7° and did not differ significantly from zero (lower 95 % CI = −1.2, upper 95 % CI = 9.2).

**Fig. 5.**
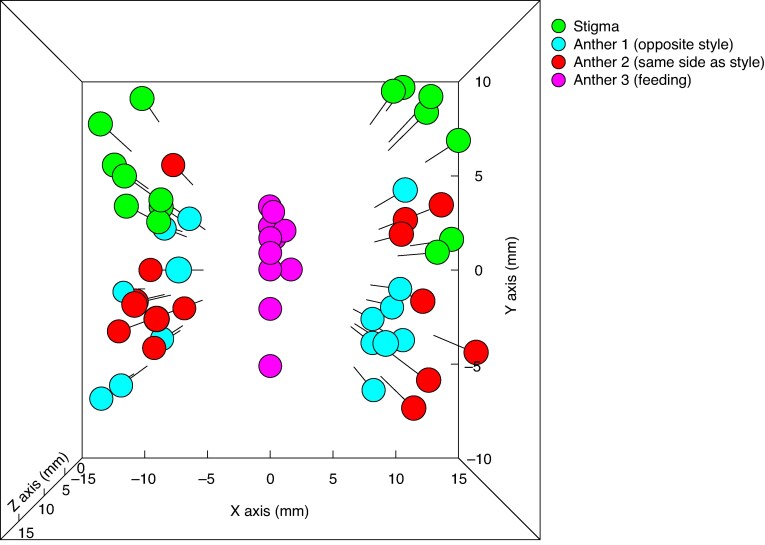
Positions of floral organs of *D. ixioides* in three-dimensional space.

The lateral anthers and style of *D. ixioides* have almost the same deflection in the *x* and *z* axis dimensions (Figs. 3, 5). However, the style is positioned slightly above the anthers in the *y* axis dimension (Fig. 5), which thus represents a form of multidimensional herkogamy. Given that the bees descend when grasping the central anther, this serves as a form of approach herkogamy that ensures stigma contact before anther contact.

In terms of the potential for pollen transfer resulting from multidimensional reciprocity of floral organs of left- and right-styled flowers, lateral anther 1, opposite the style (Fig. 1B), showed the lowest inaccuracy (3DM^2^STI = 0.69); levels of inaccuracy were much higher for lateral anther 2, which is adjacent to the style (3DM^2^STI = 3.11) and the central anther 3, which is in the centre (3DM^2^STI = 1.50). In terms of the potential for pollen transfer within the same flower type, lateral anther 2 showed the lowest inaccuracy (3DM^2^STI = 0.52), whereas lateral anther 1 (3DM^2^STI = 3.10) and the central anther 3 (3DM^2^STI = 1.54) showed higher levels of inaccuracy. Pollen transfer from both lateral anthers to stigmas is thus highly likely, whereas pollen transfer from the central anther to stigmas is very unlikely.

### Heteranthery and pollen production

The central stamen of *D. ixioides* is significantly shorter than the lateral stamens (Table 2). It also has a much larger anther and produces three times more pollen grains than do each of the lateral stamens (Table 2). However, pollen grains from the central anther were found to have almost identical dimensions to those from the lateral anthers (Table 2). As was the case for pollen production (see Methods), stamen dimensions and pollen dimensions did not differ significantly according to the stylar orientation of flowers (distance between lateral anthers: χ^2^_1_ = 1.08, *P* = 0.29; stamen length: *F*_1,28_ = 0.02, *P* = 0.89; pollen length: *F*_1,8_ = 1.50, *P* = 0.25).

**Table 2.** Measurements of traits of central and lateral stamens of *Dilatris ixioides.*

Trait	Central stamen	Lateral stamen	*F*	*P*
Stamen length (mm)	11.6 ± 0.28 (8, 8)	16.2 ± 0.28 (8, 8)	345.4	< 0.0001
Anther length (mm)	3.09 ± 0.07 (8, 8)	1.13 ± 0.07 (8, 8)	487.1	< 0.0001
Anther width (mm)	2.50 ± 0.43 (8, 8)	0.43 ± 0.05 (8, 8)	867.0	< 0.0001
Pollen number (total count method)	4114.5 ± 265.0 (10, 10)	1719 ± 111.2 (10, 10)	112.6	< 0.0001
Pollen number (subsample method)	4607.9 ± 492.8 (27, 26)	1293.1 ± 121.3 (27, 50)	81.22	< 0.0001
Pollen length (µm)	53.7 ± 0.89 (10, 46)	53.3 ± 0.88 (10, 51)	0.51	0.47
Pollen width (µm)	32.1 ± 0.37 (10, 51)	32.4 ± 0.35 (10, 57)	0.47	0.49

Values are means ± s.e. with the number of plant individuals (levels of the random effect) and samples analysed per stamen type given in parentheses. Pollen counts are per anther.

### Spectral reflectance

**Fig. 6.**
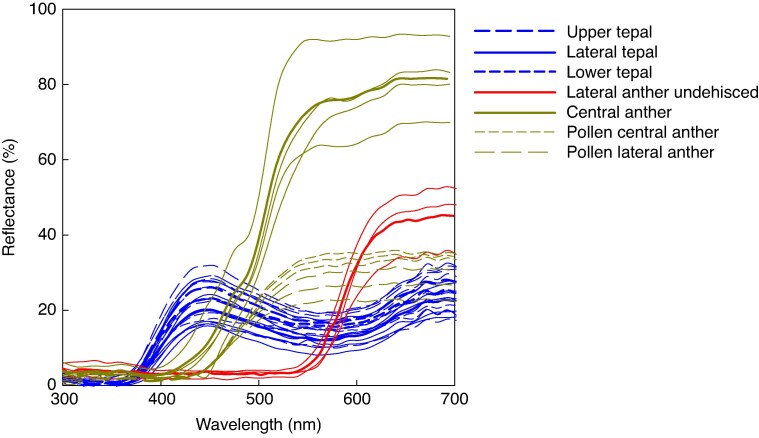
Spectral reflectance of different parts of *D. ixioides* flowers. Bold lines indicate means and feint lines are individual measurements.

Tepals of *D. ixioides* are blue/purple with minimal UV reflectance below 400 nm (Fig. 6). The central anther and pollen are both yellow and are not UV-reflecting. The central anther showed higher reflectance than did pollen, but the overall hue of the central anther was very similar to that of pollen from both central and lateral anthers. The undehisced lateral anthers are bright red in colour with no UV reflectance. After dehiscence, the lateral anther becomes inverted and appears brownish yellow.

### Stigma pollen loads and natural fruit set

*Dilatris* pollen was present on 36 (63.1 %) of the 57 stigmas (from 57 plants) that were examined. The mean pollen number of *Dilatris* pollen grains per stigma was 4.36 ± 0.63 (range 0–16) versus 0.09 ± 0.04 (range 0–5) for heterospecific pollen. The very small diameter of the stigma (Fig. 1D) means that it can only accommodate up to nine *Dilatris* pollen grains in full contact with the surface (Fig. 1E). However, we observed a cap of sticky mucilaginous exudate on the surface of many of the stigmas (Fig. 1D) and it appears that this exudate, which may be present when stigmas are receptive, allows the stigma to capture more pollen grains (up to 16 were recorded) than can fit directly on the stigmatic surface.

### Controlled pollination experiments

The mean (± s.e.) percentage of flowers that set fruit differed significantly among pollination treatments (*F*_4,344_ = 22.04, *P* < 0.001). Fruit set in naturally pollinated flowers was 79.3 ± 4.4 %, which was significantly greater (*P* < 0.001, Sidak post-hoc test) than that in bagged unmanipulated flowers (6.7 ± 2.9 %), flowers self-pollinated using lateral anthers (7.0 ± 4.0 %), flowers cross-pollinated using the central anther (7.9 ± 4.5 %) and flowers cross-pollinated using the lateral anthers (17.3 ± 6.3 %). Similarly, seed set as a percentage of available ovules in flowers (including those that did not set fruit) differed significantly among treatments (*F*_4,377_ = 27.3, *P* < 0.001). The value for naturally pollinated flowers was 32.6 ± 2.3 %, which was significantly greater (*P* < 0.001, Sidak post-hoc test) than the values for bagged unmanipulated flowers (3.3 ± 1.0 %), flowers self-pollinated using lateral anthers (4.1 ± 1.9 %), flowers cross-pollinated using the central anther (4.3 ± 1.9 %) and flowers cross-pollinated using the lateral anthers (8.1 ± 2.6 %). In the post-hoc tests there were no significant differences among cross-pollinated and self-pollinated flowers in terms of either fruit set or the percentage of ovules that developed into seeds.

## DISCUSSION

### Functions of enantiostyly and heteranthery for wing pollination

Flowers of *Dilatris ixioides* have a novel combination of features that results in a finely tuned system of wing pollination by pollen collecting bees. The lateral stamens and style are deflected away from the centre of the flower so that the lateral anthers and stigma (positioned alternately to the left or right) are positioned to make contact with the beating wings of large bees. The lateral anthers are small and red, making them less conspicuous to bees which have poor perception of red wavelengths (Lunau et al., 2011). Nectar is absent and the centre of the flower is occupied by a single large central anther with spectral reflectance similar to pollen and also produces far more pollen than the lateral anthers. This large anther acts as a super-stimulus for grabbing the attention of pollen-collecting bees and positioning them in the centre of the flower. Thus, enantiostyly and heteranthery function synergistically in this wing pollination system.

Enantiostyly is widespread in the Haemodoroideae and was almost certainly an antecedent condition in the lineage that led to *Dilatris*. Pronounced deflection of the style and stamens in Haemodoroideae appears to be associated with various systems of wing pollination (Minnaar and Anderson, 2021; Johnson et al., 2023). Enantiostyly most likely evolved gradually from straight-styled ancestors and the initial evolution of enantiostyly would have been characterized by moderate deflection of the style to maximize pollen receipt from lateral parts of the pollinator’s thorax and abdomen, as has been recorded in many species of Solanaceae and Fabaceae (Bowers, 1975; Dulberger, 1981). Many enantiostylous plant species with moderate deflection of the style are buzz-pollinated and because bees usually fold their wings when sonicating flowers (Bowers, 1975; Buchmann, 1983), these plants are most likely to utilize the thorax and abdomen of bees, rather than their wings, for pollen transfer. Buzz-pollination is thus likely to constrain the evolution of wing pollination. The evolution of widely diverging styles and stamens with a wing pollination function appears to be linked with floral traits, such as the absence of a landing platform, that ensure that pollinators are kept hovering while utilizing rewards.

In *Wachendorfia* and *Barberetta*, hovering flower visitors feed on nectar in the centre of the flower while transferring pollen from the laterally deflected anthers to the laterally deflected style with their wings. It is more complex to achieve wing-mediated transfer of pollen via precise pollinator positioning when pollen is the reward. In *D. ixioides* this problem (of needing to bring the flower visitor into a central position) is overcome by deploying a single stamen with a large feeding anther in a central position and two lateral stamens that are positioned in a horizontal plane to contact the beating wings of insect visitors.

In *D. ixioides* enantiostyly clearly functions as an effective mechanism for transfer of pollen on the wings of pollen-collecting bees, particularly *X. rufitarsus* which shows excellent fit to the flowers in terms of the match of its wingspan to the lateral anthers and the stigma (Fig. 3). Given that the wings of these bees beat against the stigmas and anthers for ∼477 ms and the wingbeat frequency of carpenter bees is ∼125 Hz (Roberts et al., 2004), we estimate that the lateral anthers and the stigma of *D. ixioides* are, on average, struck approximately 60 times by the wings of each visiting carpenter bee. This helps to explain how the minute stigma picks up pollen grains scattered across the wings of the bees.

A key element of the wing pollination system of *Dilatris*, like that of *Wachendorfia* (Minnaar and Anderson, 2021) and *Barberetta* (Johnson et al., 2023), is the horizontal orientation of the flowers across the plane of the lateral anthers. This was misunderstood by Simpson (1990, p. 754), who characterized the flowers of *Dilatris* as actinomorphic and erect and lacking bilaterally symmetric nectar guides, and thus concluded that visitors ‘would be positioned inconsistently with regard to the deflected style’. Simpson (1990, p. 754) further argued that ‘enantiostyly in [*Dilatris*, *Barberetta*, *Lachnanthes* and *Xiphidium*] may be adaptive in simply decreasing the chance of self-pollination by physically displacing the stigma from anthers’. From this study of *D. ixioides* and our previous study of *Barberetta aurea* (Johnson et al., 2023), we now know that Simpson’s interpretation was incorrect and that enantiostyly in these two taxa is associated with a very precise system of wing pollination.

The reciprocity of anthers and stigmas among and within flower types of *D. ixioides* can only be understood in terms of a three-dimensional analysis. The central stamen of *D. ixioides* clearly functions for reward provisioning and has a very low level of spatial reciprocity with the stigma, both in the *x* axis because it is positioned centrally and in the *z* axis as it has a filament that is shorter than those of the lateral stamens (Fig. 5). The lateral anthers show reciprocity with the stigma, particularly in the *x* axis dimension, both among flower types in the case of anther 1 and within flower types in the case of anther 2 (Fig. 5). However, the reciprocity is not perfect in the *y* axis dimension as the stigma is positioned above the horizontal plane of these lateral anthers (Fig. 5). There are two obvious reasons for this floral architecture. First, perfect three-dimensional reciprocity of anther 1 with the stigma of the same flower type would result in absence of herkogamy and thus autonomous self-pollination. Second, bees settle downwards on the flower and grasp the central anther, which has a flimsy filament that does not support their weight, and this downward movement results in the first contact (associated with pollen deposition) being between the wing and the stigma, whereas the second contact (associating with pollen loading on the insect) is between the wings and the lateral anthers. This is effectively a type of approach herkogamy (physical separation of the stigma and anthers such that an approaching animal contacts the stigma before the anthers).

### Floral signals

The spectral features of *D. ixioides* are consistent with other flowers that are pollinated by pollen-collecting bees. The combination of a blue corolla and a central yellow target, either in the form of guide patterns on the corolla or the anthers, is the most common form of visual advertising in pollen-rewarding plants (Lunau, 1991, 1995; Lunau et al., 2006; Russell et al., 2016). The blue colour is known to elicit innate attraction of bee species and the main floral ‘target’ in the form of the large yellow central anther and pollen is a feature that stimulates innate responses by bees (Lunau, 1991; Muth et al., 2016; Lunau et al., 2024). Consistent with work that has been conducted on other bee groups (Lunau, 1991, 1992; Lunau et al., 2024), the carpenter bees in this study touch the central yellow target with their antennae. This behaviour likely involves olfaction and it is likely that pollen-rewarding systems, such as the one in *D. ixioides*, have to be at least partially honest in the sense that bees would reject further exploration of anthers that do not have the smell (and possibly the feel) of sufficient pollen grains. We observed that bees sometimes stroked the central anther with their antennae and then flew away without grasping the anther, presumably because the anther did not provide a sensory stimulus that is consistent with large numbers of pollen grains.

It is now well-established that bees can detect pollen odour (Dobson et al., 1999; Carr et al., 2015). It has also been shown that bees can learn to associate flower features such as colour and target patterns with pollen rewards. This has been shown both using artificial flowers (Muth et al., 2015; Nicholls et al., 2015; Nicholls and Hempel de Ibarra, 2017) and real flowers (Russell et al., 2016). Some of these studies have been conducted using honeybee-collected pollen, which contains nectar from sugars, but Muth et al. (2016) showed that bees can make these associations even when collecting flower-collected pollen that had not been mixed with nectar (and nectar-based volatiles). The flowers of *D. ixioides* would only need to elicit some initial attraction of carpenter bees through their innate response to particular cues, whereupon they would quickly learn to associate the copious pollen rewards with the visual signals of the flowers.

### Pollinator dependence

The controlled pollination experiments showed that fruit set of *D. ixioides* is reduced dramatically about ten-fold from ∼80 % to ∼8 % when pollinators are excluded from flowers. We did, however, record some fruit and seed set in the unmanipulated bagged flowers and this did not differ significantly from the values for hand-pollinated flowers. Our interpretation of the fruit and seed set in the bagged treatments is that *D. ixioides* is partially self-compatible, as is the case for related genera such as *Wachendorfia* and *Barberetta* (Jesson and Barrett, 2002a; Johnson et al., 2023). The fruits and seeds that we recorded in the bagged unmanipulated flowers likely arose from a ‘bag effect’ whereby the action of the wind rubs the bag over the highly exserted anthers and stigmas of the flowers, resulting in self-pollination. Flowers of *D. ixioides* have strong herkogamy and there is no anther–stigma contact as the flowers do not close up after anthesis, thus ruling out autonomous self-fertilization. The low fruit and seed set in the cross-pollination treatment relative to naturally pollinated flowers is likely to be a reflection of unsuccessful hand pollinations, possibly due to mismatch in the timing of the hand-pollinations and stigmatic receptivity. Due to the very small diameter of the stigma (∼0.15 mm) we were unable to determine if the stigmas that we hand-pollinated had the cap of mucilage (Fig. 1D, E) that likely retains pollen and promotes germination or were immature or had dried out. However, from the results obtained it is possible to conclude that *D. ixioides* is largely pollinator-dependent and at least partially self-compatible.

The large floral displays of *Dilatris* species are likely to result in some geitonogamous pollen transfer (Barrett et al., 2000), although we found that bees visited only ∼20 % of the flowers that were open on each plant of *D. ixioides*. Although we were unable to determine if self-pollination in *D. ixioides* has deleterious consequences for female fecundity, it is highly likely to lead to some pollen discounting (loss of male siring opportunities due to pollinator-mediated self-pollination). Experiments by Jesson and Barrett (2002b) using allozyme markers for buzz-pollinated *Solanum rostratum*, which has a single pollinating anther, have shown that monomorphic enantiostyly can reduce overall levels of pollinator-mediated selfing in plants, but not to the same extent as dimorphic enantiostyly. However, the symmetrical arrangement of the lateral stamens on both sides of *Dilatris* flowers, with equal loading of pollen onto both wings of pollinators, is likely to limit the effectiveness of enantiostyly for reduction of geitonogamy. Wing pollination and geitonogamy reduction can be complementary functions of enantiostyly and their relative importance in the original evolution of this trait syndrome is likely to differ among plant lineages depending on their floral architecture and reward system.

### Conclusions

This first study of pollination in the genus *Dilatris* reveals a novel system of pollen reward and pollen transfer on the wings of pollen-collecting bees. The two most important features of the floral architecture of this system are heteranthery (an oversized central anther that bees focus on for pollen collection and two laterally deflected anthers that place pollen on the wings) and enantiostyly (deflection of the style either to the left or to the right, such that the stigma is placed to receive pollen from the beating wings of the bees). Given that there is variation in the degree of heteranthery and enantiostyly in *Dilatris*, it would be of interest to examine the pollination systems of other species in the genus.

Additional case studies of floral traits and pollination systems in other genera of the Haemodoroideae will be required to reconstruct the history of floral evolution in this clade. The degree of enantiostyly is clearly variable, and sometimes even barely discernible, within genera in the subfamily, and although strong alternating style deflection in *D. ixioides* has a clear function for wing pollination and results in flower handedness, it is much less clear whether this is the case in species with weak style deflection, such as *Xiphidium caeruleum*, which is buzz-pollinated and has a style that is only slightly curved and occupies a central position in the flower surrounded by three stamens with similar anthers (Buchmann, 1980). Although data on nectar production are incomplete, available evidence suggests that nectar production varies among genera in the Pontederiaceae, which is sister to Haemodoraceae, and is ubiquitous in the Conostylidoideae, which is the sister clade to Haemodoroideae that includes *Dilatris*. In the Haemodoroideae, nectar production occurs in several genera including *Haemodorum*, *Wachendorfia* and *Barberreta* (Simpson, 1993). *Dilatris* and *Xiphidium* lack nectar but are not closely related to each other. It therefore seems very likely that nectar production was lost twice in the Haemodoroideae and in both cases this was associated with anther modifications that enable pollen to be deployed as the sole reward.

More broadly, this study also identifies floral features that force insects to collect rewards while hovering as a key factor in the evolution of widely diverging styles and stamens with a wing-pollination function. This could explain why wing pollination appears to be rare among enantiostylous plants that are buzz-pollinated by bees that fold their wings when sonicating flowers.

## Supplementary Material

mcaf189_Supplementary_Data

## SUPPLEMENTARY DATA

Supplementary data are available at *Annals of Botany* online and consist of the following. Figure S1. Screen grabs from slow-motion videos showing sequence of behaviour of *Xylocopa rufitarsus* bees on flowers of *D. ixioides*. Figure S2. The inflorescence architecture of *Dilatris ixioides*. Video S1. Wing pollination of *Dilatris ixioides* by pollen-collecting carpenter bees.
