# Supplementary material for: Functions of heteranthery and enantiostyly for wing pollination by pollen-collecting bees in Dilatris ixioides (Haemodoraceae)
Source: Ann Bot. 2025 Aug 23;137(3):641–53. doi: 10.1093/aob/mcaf189 (PMC12933676; doi:10.1093/aob/mcaf189)
Supplement: mcaf189_Supplementary_Data [file mcaf189_supplementary_data.zip › Dilatris supplemental material.docx]

SUPPLEMENTAL MATERIAL

Functions of heteranthery and enantiostyly for wing pollination by pollen-collecting bees in *Dilatris ixioides* (Haemodoraceae)

Steven D. Johnson, Jeremy J. Midgley & Nicola Illing


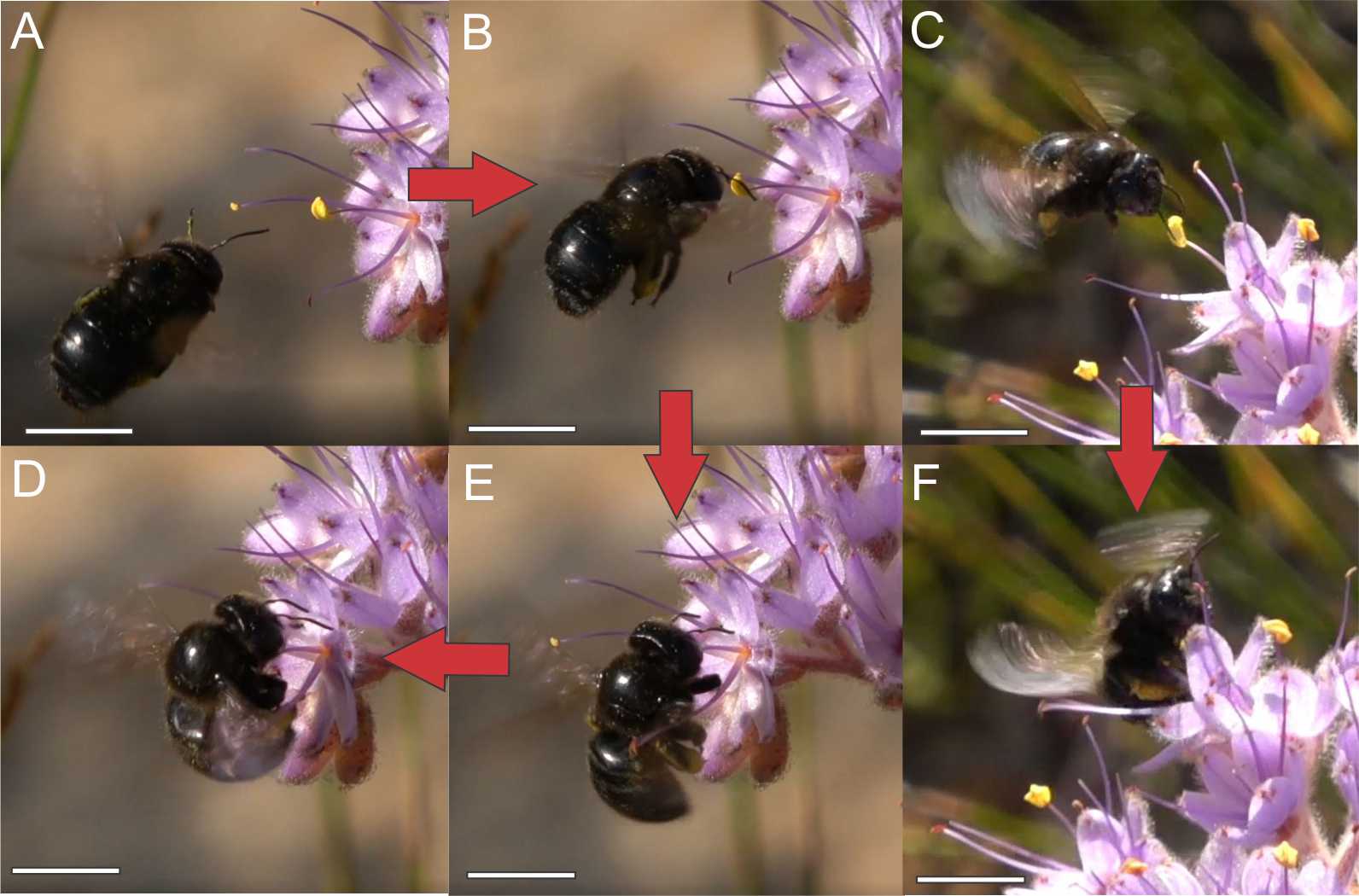


Fig S1. Screen grabs from slow-motion videos showing sequence of behaviour of *Xylocopa rufitarsus* bees on flowers of *D. ixioides*. A. Approach. B-C. Antennating on central anther. D-F. Grasping central anther while wings beat over stigma and lateral anthers. Scale bars = 10 mm.


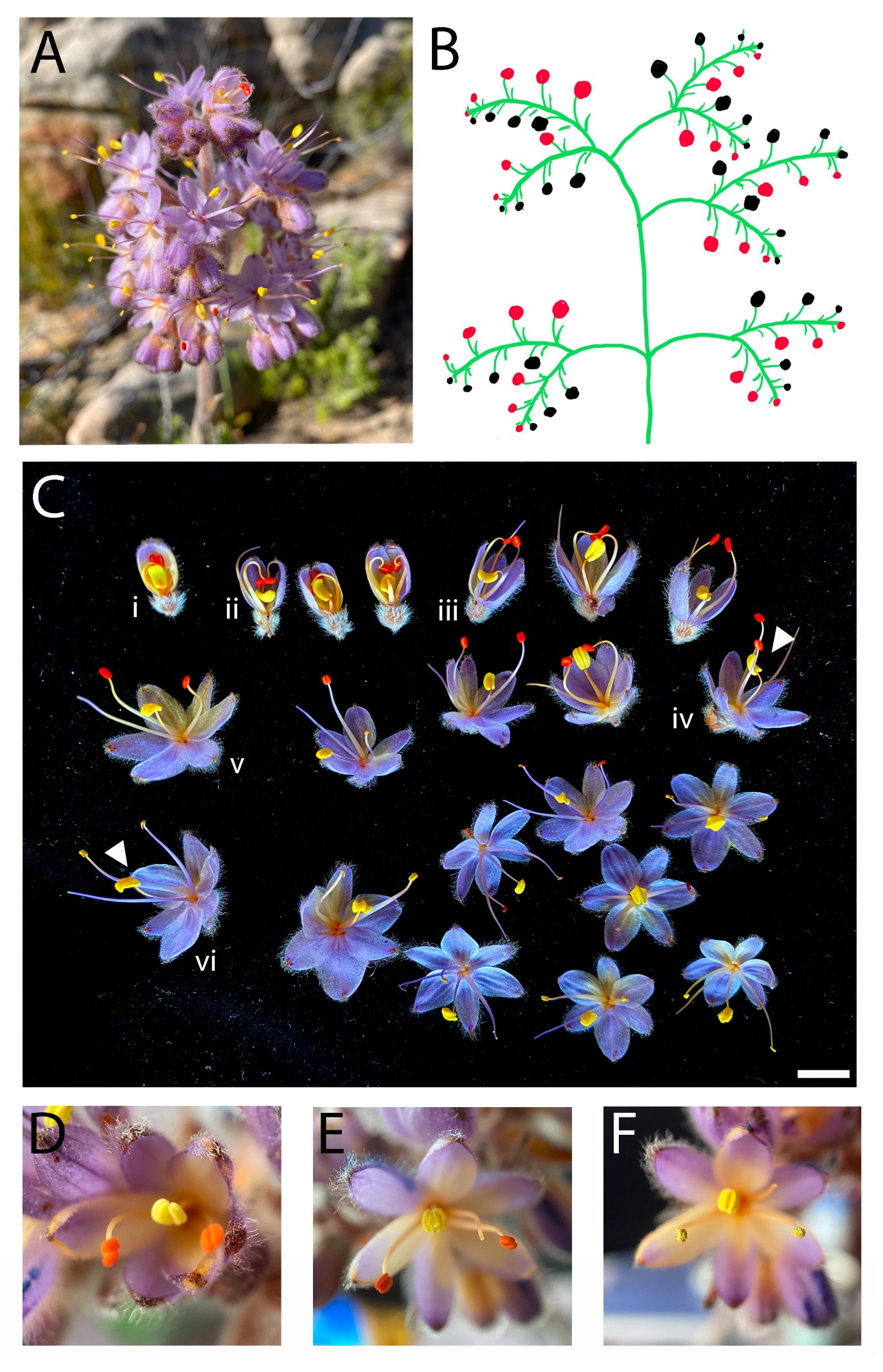


Fig S2. The inflorescence architecture of *Dilatris ixiodes*. A).Stems are twisted 180˚ between alternating flowers, such that one side of the stem has flowers with styles in one direction and the other side has flowers with styles in the opposite direction. Styles are deflected towards the axis of the cyme. B) Inflorescences are a bifurcating or trifurcating helicoid cyme. The inflorescence has pendulum symmetry with alternately right- (●) or left-(●) handed flowers on each branch. C) Sequence of flower development and maturation, moving from i) to vi). The red lateral anthers are initially folded over the yellow central anther (i) and elongate rapidly together with the style as the bud develops (ii) emerging from the bud prior to the flower opening (iii). The yellow central anther (indicated by a white triangle) reorientates itself by 180^o^ as the open flower matures (iv to v). The red colour of the lateral anthers becomes less obvious after pollen dehiscence (vi). Scale bar = 10mm. Dashed red lines group flowers of similar developmental stages. D-F) Order of anther dehiscence.  The central anther matures first (E), followed by the lateral anthers (F).
